# Supplementary material for: Social networks and risk of delayed hospital arrival after acute stroke
Source: Nat Commun. 2019 Mar 14;10:1206. doi: 10.1038/s41467-019-09073-5 (PMC6418151; doi:10.1038/s41467-019-09073-5)
Supplement: Supplementary file 1 — Supplementary Information [file 41467_2019_9073_MOESM1_ESM.pdf]

Social Networks and Risk of Delayed Hospital Arrival after Acute Stroke

Dhand et al.

Supplementary Table 1: Context and Communication Patterns of Slow and Fast Arrivers

**Slow Arrivers**	
Social context at time of stroke	*Description of communication*
1. With husband and grandson (strong ties) and then husband called daughter (strong tie).	Patient did not tell family initially. Patient argued with her husband when she could not move. Husband agreed to wait. Daughter called the ambulance after she returned from work.
2. With niece (strong tie).	He did not tell niece initially. He wanted to wait and "see what happens first." When situation did not change, he asked niece to call 911.
3. With husband (strong tie) and called sister (strong tie).	Husband and sister did not think symptoms were serious and confirmed each other's opinions. Patient decided to call ambulance herself.
4. Called close friend (strong tie).	Patient waited to call friend initially because he did not want to bother him at 5:00am. Friend drove him to the hospital eventually.
5. Called close friend (strong tie).	Patient did not tell friend or other family initially because telling them caused him stress. When he did tell them, they told him to go to the hospital and he declined. Eventually, he called 911.
6. With close friend (strong tie) and called son (strong tie).	Patient did not tell close friend initially. He called son who called an ambulance.
7. With wife (strong tie).	Patient and wife negotiated symptoms. Patient convinced wife to drive instead of call 911.
8. With coworkers (weak ties) and then daughter and husband (strong ties).	Patient did not tell coworkers. She told husband and daughter who advised to get help and she declined. She then drove herself to hospital.
9. With doctor (weak tie) and then nephew (strong tie). Patient called close friend (strong tie) and then called aunt (weak tie).	Patient explained symptoms to doctor who did not think symptoms were serious. He did not tell nephew or close friend initially and then negotiated plan to wait. His Aunt, a nurse, thought symptoms were concerning and advised calling ambulance.
10. With daughter (strong tie).	Daughter called 911 when patient slid off toilet.
11. With son and husband (strong ties).	Son and husband decided on action plan without asking patient.
12. Alone.	Patient fell on the floor and then called 911 herself when she reached the phone.
**Fast Arrivers**	
Social context at time of stroke	*Description of hospital arrival process*
1. With coworker (weak tie) who called his supervisor (weak tie).	Coworkers noticed abnormal behaviors and called ambulance without discussing with patient.
2. With acquaintance (weak tie).	A staff member called 911 when the patient fell and could not get up during a class.

3. With friends (weak ties).	Patient was playing basketball with friends. Two friends noticed signs and one friend drove patient to the hospital.
4. With dentist and receptionist (weak ties).	Dentist recognized stroke signs and insisted that patient (who was reluctant) go to the hospital by ambulance. Receptionist called 911.
5. With coworkers (weak ties) and son (strong tie) and called his wife (strong tie).	Coworkers and wife determined that the patient should go to the hospital immediately. Son drove him from work to the hospital.
6. With house guest (weak tie) and wife (strong tie).	House guest recognized unilateral weakness and told wife. The wife drove him to the hospital.
7. Alone and then with nurse (weak tie).	Patient, a former nurse, "diagnosed myself" and then called nurse who called 911.
8. With stranger in public (no tie).	Patient fell backwards in public, and a stranger called 911 without asking her.
9. Alone.	Patient had nausea and vomiting and decided to go to the ER himself. He did not call anyone.
10. Alone.	Patient did not discuss with anyone and called 911.
11. With husband (strong tie).	Husband noticed the patient was disoriented and called the ambulance.
12. With wife (strong tie).	Patient argued with his wife, but she called the ambulance anyway. "Her decision, her choice, her intervention."
13. With husband (strong tie).	Patient and husband decided that patient needed an ambulance, and husband called 911.
14. Called fiancé (strong tie).	Patient and fiancé decided that the patient needed to go to the hospital. Fiancé drove her.
15. Called daughter (strong tie).	Patient fell and called daughter. Daughter asked her husband to call ambulance.

Supplementary Figure 1: Sensitivity Analysis, Delay defined as greater than 3 hours.

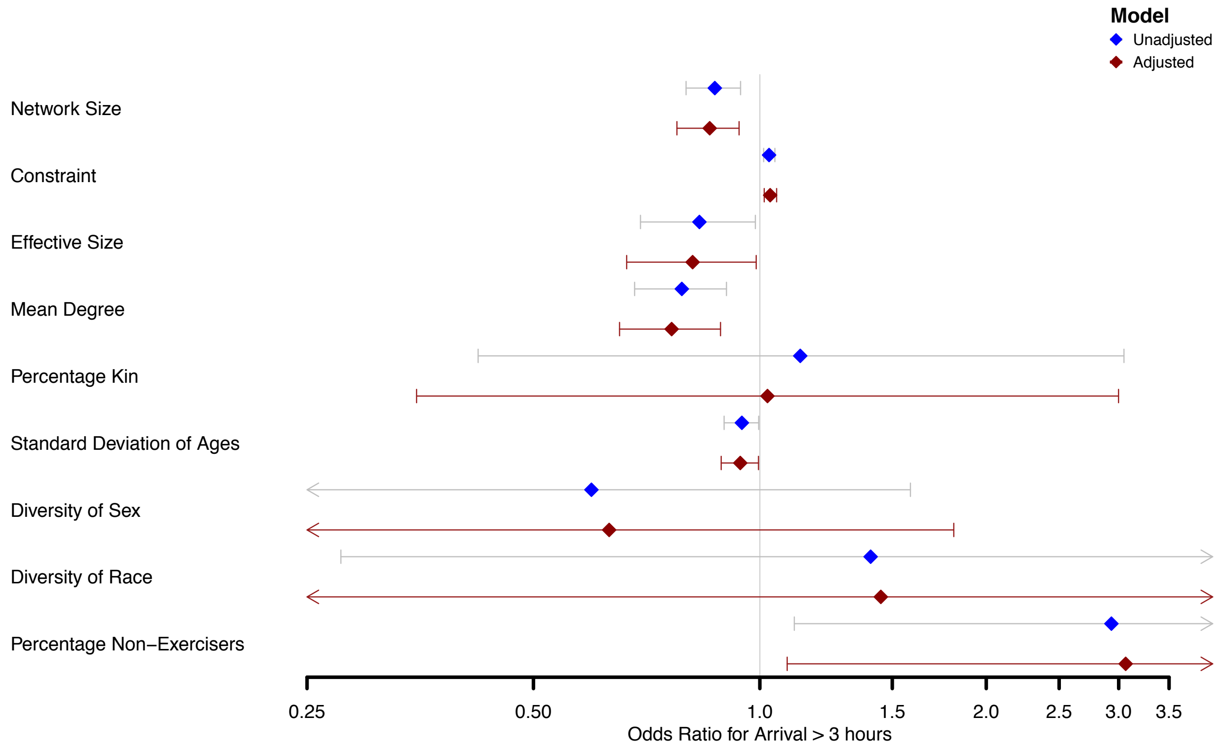

Blue diamonds are unadjusted Odds Ratio with 95% confidence intervals. Red diamonds are Odds Ratio with 95% confidence intervals adjusted for age, stroke severity, black race, education, median income, living alone, and use of emergency medical services.

Supplementary Figure 2: Sensitivity Analysis, Removal of networks smaller than 5.

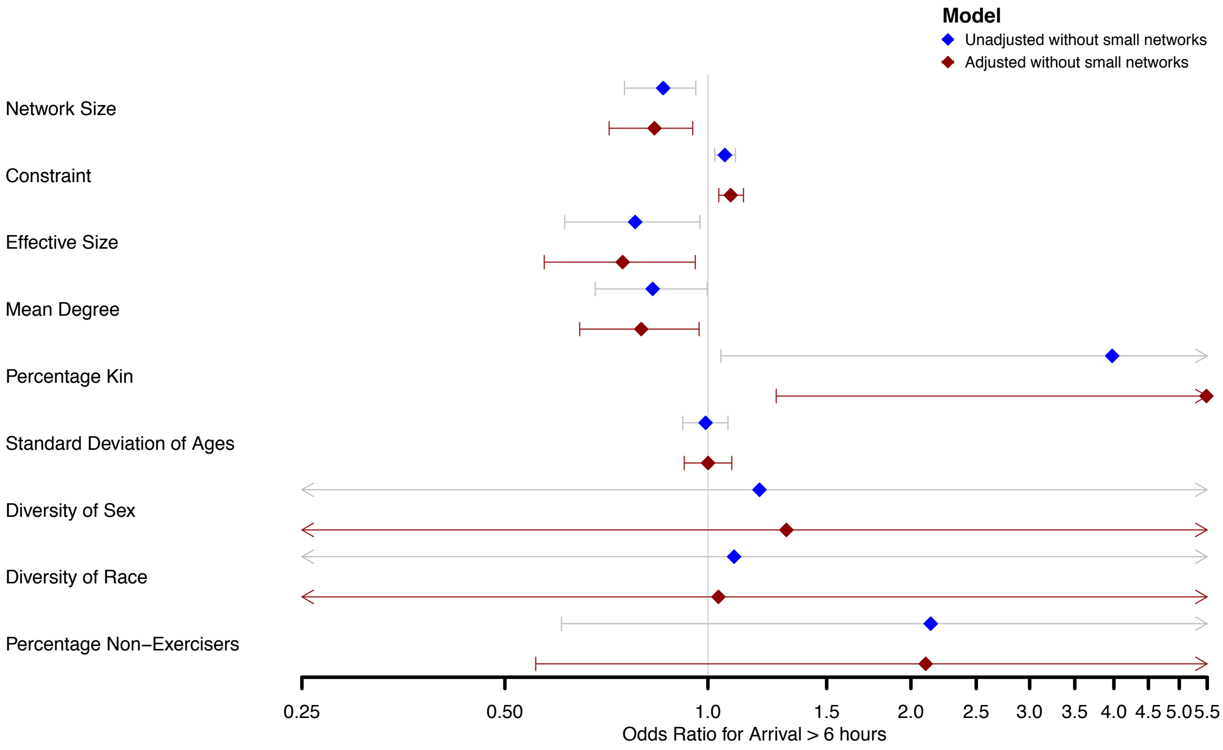

Delay is defined as greater than 6 hours. Blue diamonds are unadjusted Odds Ratio with 95% confidence intervals. Red diamonds are Odds Ratio with 95% confidence intervals adjusted for age, stroke severity, black race, education, median income, living alone, and use of emergency medical services.

Supplementary Figure 3: Sensitivity Analysis, Only Persons who are Black/African American.

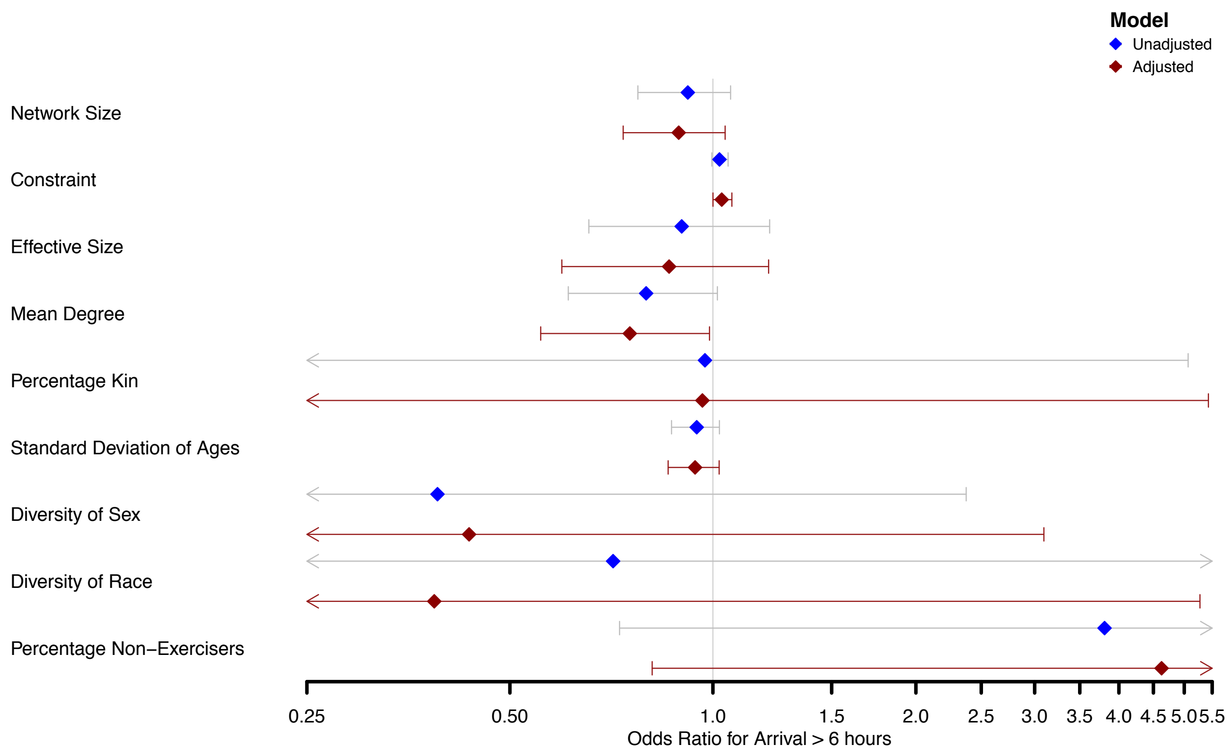

Delay is defined as greater than 6 hours. Blue diamonds are unadjusted Odds Ratio with 95% confidence intervals. Red diamonds are Odds Ratio with 95% confidence intervals adjusted for age, stroke severity, education, median income, living alone, and use of emergency medical services.

Supplementary Figure 4: Sensitivity Analysis, Only Persons who are Not Black/African American.

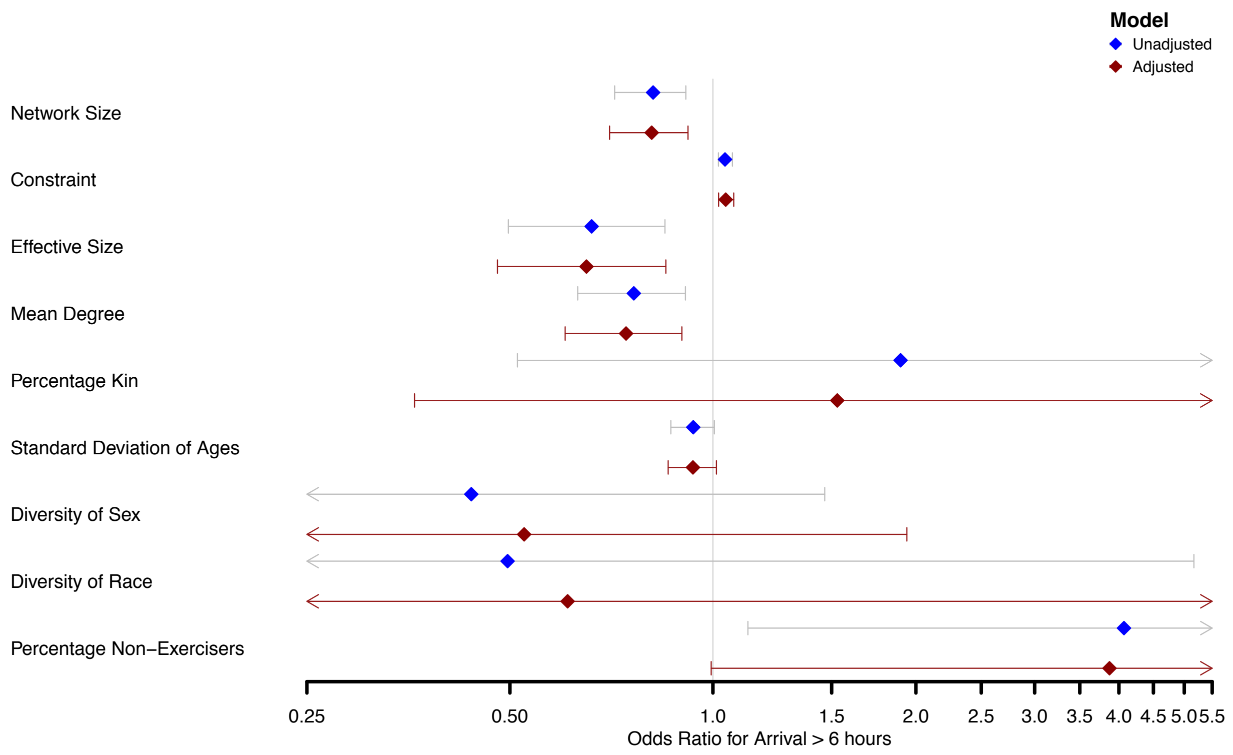

Delay is defined as greater than 6 hours. Blue diamonds are unadjusted Odds Ratio with 95% confidence intervals. Red diamonds are Odds Ratio with 95% confidence intervals adjusted for age, stroke severity, education, median income, living alone, and use of emergency medical services.

Supplemental Methods 1: Social Network Assessment Instrument

Q1. From time to time, most people discuss important personal matters with other people. Looking back over the last 3 months, who are the adults with whom you discussed an important personal matter? Please just tell me their first names or initials.

Q2. From time to time, people socialize with other people. For instance, they visit each other, go together on a trip or to a dinner. In the last 3 months, who are the adults with whom you usually do these things? Please just tell me their first names or initials. Don't worry about duplicates. We will deal with them after you are done.

If there are duplicates in Q1 and Q2 Names:
Is [NAME] the same as [NAME] who you listed before?

If fewer than five names are given:
Anyone else?

RESPONDENT										
EC	NAME 1									
EC	S EC	NAME 2								
EC	S EC	S EC	NAME 3							
EC	S EC	S EC	S EC	NAME 4						
EC	S EC	S EC	S EC	S EC	NAME 5					
EC	S EC	S EC	S EC	S EC	S EC	NAME 6				
EC	S EC	S EC	S EC	S EC	S EC	S EC	NAME 7			
EC	S EC	S EC	S EC	S EC	S EC	S EC	S EC	NAME 8		
EC	S EC	S EC	S EC	S EC	S EC	S EC	S EC	S EC	NAME 9	
EC	S EC	S EC	S EC	S EC	S EC	S EC	S EC	S EC	S EC	NAME 10

Q2 Names:

______ ______
______ ______
______ ______
______ ______
______ ______

IF GREATER THAN 10, RECORD TOTAL NUMBER OF PEOPLE NAMED IN THIS BOX.

Q3. Compared to the others you mentioned, do you feel especially close to [NAME 1]? Do you feel especially close to [NAME 2]? *Repeat for each name.*

Q4. I will now focus on the relations between each pair of people you mentioned. For example, I will ask about [Name 1] and [Name 2]. For each pair, I will ask whether they are total strangers, especially close, or in-between. By total strangers, I mean that they wouldn't recognize one another if they met on the street. By especially close, I mean as close to each other as they are to you. By in-between, I mean all other relationships. Do you understand?

Is [NAME 1] a total stranger, especially close, or in-between with [NAME 2]? *Repeat for each name.*

Q5. I'm now going to ask about how much support you have from people in general. If you needed it, how often is someone available…

A. to help you if you were confined to bed?

1. None of the time
2. A little of the time
3. Some of the time
4. Most of the time
5. All of the time

☐

B. to take you to the doctor if you needed it?

1. None of the time
2. A little of the time
3. Some of the time
4. Most of the time
5. All of the time

☐

C. to prepare your meals if you are unable to do it yourself?

1. None of the time
2. A little of the time
3. Some of the time
4. Most of the time
5. All of the time

☐

D. to help with daily chores if you were sick?

1. None of the time
2. A little of the time
3. Some of the time
4. Most of the time
5. All of the time

☐

E. to turn for suggestions about how to deal with a personal problem?
1. None of the time
2. A little of the time
3. Some of the time
4. Most of the time
5. All of the time

F. who understands your problems?
1. None of the time
2. A little of the time
3. Some of the time
4. Most of the time
5. All of the time

G. to love and make you feel wanted?
1. None of the time
2. A little of the time
3. Some of the time
4. Most of the time
5. All of the time

Q6. Out of the people you've mentioned before [*Repeat All Names*], which person or persons do these supportive actions most often? You can choose more than one person.

1. YES
2. NO

N1 N2 N3 N4 N5 N6 N7 N8 N9 N10

Are there any other people not mentioned who do these supportive actions?

1. Yes
2. No

If additional people are named, mark them as "Yes" in Q6. Then, go back to first page, and add to the matrix, and repeat Q3 and Q4 for new names.

Q7. I am now going to ask you a little more about each person. Does [NAME 1] have a negative influence on your health? For example does he or she passively or actively encourage you to smoke, not eat well, or not exercise? And [Name 2]? **...**

1. Yes
2. No

N1	N2	N3	N4	N5	N6	N7	N8	N9	N10

Q8. Is [NAME 1] a male or female? And [NAME 2]? **...**

1. Male
2. Female

N1	N2	N3	N4	N5	N6	N7	N8	N9	N10

Q9. Is [NAME 1] White, Black, Asian, Hispanic, or another race? And [NAME 2]? ...

1. Asian
2. Black
3. Hispanic
4. White
5. Other
6. Refused
9. Don't know

N1	N2	N3	N4	N5	N6	N7	N8	N9	N10

Q10. As far as you know, what is [NAME 1]'s highest level of education? And [NAME 2]? ...

1. 1 to 8 years
2. Some high school
3. High school grad
4. Some college
5. Associate degree
6. Bachelor's degree
7. Graduate degree
8. Don't know

N1	N2	N3	N4	N5	N6	N7	N8	N9	N10

Q11. On average, do you speak with [NAME 1] almost every day, at least once a week, at least once a month, or less than once a month? And [NAME 2]? ...

1. Daily
2. Weekly
3. Monthly
4. Less often
9. Don't know

N1	N2	N3	N4	N5	N6	N7	N8	N9	N10

Q12. Have you known [NAME 1] for less than three years, three to six years, or more than six years? And [Name 2]? …

1. Less than three
2. Three to six
3. More than six
9. Don't know

N1	N2	N3	N4	N5	N6	N7	N8	N9	N10

Q13. I'm going to give you a list of some of the ways in which people are connected to each other. Some people can be connected to you in more than one way. For example, a man could be your brother and he could belong to your church and be your lawyer. When I read you a name, please tell me all the ways that person is connected to you. How is [NAME 1] connected to you? And [NAME 2]? …

1. Spouse
2. Parent
3. Sibling
4. Child
5. Other family
6. Coworker
7. Comember in org.
8. Neighbor
9. Friend
10. Advisor
11. Other
99. Don't know

N1	N2	N3	N4	N5	N6	N7	N8	N9	N10

Q14. Has [NAME 1] ever had a stroke? And [NAME 2]? … *Skip to Q16 if no strokes.*

1. Yes
2. No
9. Don't know

N1	N2	N3	N4	N5	N6	N7	N8	N9	N10

Q15. When did [NAME] have a stroke? More than 1 year ago, between 3 months and 1 year, or within the last 3 months? …

1. More than 1 yr
2. 3 mo and 1 yr
3. Last 3 mo
9. Don't know

N1	N2	N3	N4	N5	N6	N7	N8	N9	N10

Q16. How old is [NAME 1]? What is your best guess? And [NAME 2]? …

1. Number of years
8. Refused
9. Don't know

N1	N2	N3	N4	N5	N6	N7	N8	N9	N10

Q17. Have you done any of the following to try and improve your health in the past 3 months?

A. Cut back smoking?

1. Yes
2. No
9. Not applicable

B. Exercised at least 3 times per week?

1. Yes
2. No
9. Not applicable

C. Regularly taken your medications, meaning missed them less than once per week?

1. Yes
2. No
9. Not applicable

D. Attended all doctor appointments?

1. Yes
2. No
9. Not applicable

Q18. Has [NAME 1] done any of the following to try and improve his or her health in the past 3 months. And [NAME 2]? ... *Go down the column for each Name before moving to next.*

A. Cut back smoking?

1. Yes
2. No
8. Don't know
9. Not applicable

N1 N2 N3 N4 N5 N6 N7 N8 N9 N10

B. Exercised at least 3 times per week?

1. Yes
2. No
8. Don't know
9. Not applicable

N1 N2 N3 N4 N5 N6 N7 N8 N9 N10

C. Regularly taken his or her medications, meaning missed them less than once per week?

1. Yes
2. No
8. Don't know
9. Not applicable

N1 N2 N3 N4 N5 N6 N7 N8 N9 N10

D. Attended all doctor appointments?

1. Yes
2. No
8. Don't know
9. Not applicable

N1 N2 N3 N4 N5 N6 N7 N8 N9 N10

Q19. How far away does [NAME 1] live? And [NAME 2]? …

1. 50+ miles
2. 16-50 miles
3. 6-15 miles
4. 1-5 miles
5. Same house

N1	N2	N3	N4	N5	N6	N7	N8	N9	N10

Notes of remarkable elements of the interview:

Demographics and stroke information

Occupation:

Education level:

Marital status:

Does participant live alone?:

Did the participant use EMS to arrive at the ER?:

Are family members present during the interview?:

Hours to arrival (key cut points are < or > 3 hours and < or > 6 hours; confirm with patient if unclear):

Obtain these demographics from medical chart:

Patient name:

Date of birth:

Gender:

Race:

Ethnicity (Hispanic or Latino, NOT Hispanic or Latino, Unknown):

NIHSS:

Stroke location (Left/Right):

Area with majority of damage (Cortical/Subcortical/Brainstem/Cerebellum):

Aphasia (Yes/No):

tPA used:

Disposition:

Short Blessed Score:

Charlson Score (Based on Past Medical History):

CARD Q10

Spouse: Your wife, your husband, or a person with whom you are living as if married.

Parent: Your father or mother.

Sibling: Your brother or sister.

Child: Your son or daughter.

Other family: For example, grandparent, grandchild, cousin, aunt, uncle, nephew, niece, or an in-law.

Coworker: Someone who works with you, or usually meet while working.

Co-member in organization: Someone who is a member of the same organization as you. For example, a person who attends the same church, attends the same school, or belongs to the same club.

Neighbor: Someone outside your own household who lives close to you in your neighborhood.

Friend: Someone who you get together with for informal social occasions. This includes a boyfriend or girlfriend.

Professional advisor or consultant: A trained expert who gives you advice. For example, a lawyer or a clergyman.

Other

Supplemental Methods 2: Equations for Social Network Metrics

(1) $$\text{Size} = N$$

where N is the number of nodes, not including the ego.

(2) $$\text{Density} = \frac{2L}{N(N-1)}$$

where L is the number of ties, and N is the number of nodes, not including the ego.

(3) $$\text{Constraint of i's network} = (p_{ij} + \sum_q p_{iq} \times p_{qj})^2$$

where i is the ego, q and j are alters, p_{ij} is the proportional strength of i's relation with j, p_{iq} is the proportional strength of i's relation with q, and p_{qj} is the proportional strength of q's relation with j.

(4) $$\text{Effective size of } i\text{'s network} = \sum_j \left[1 - \sum_q p_{iq} \times m_{jq} \right], q \neq i, j$$

where i is the ego, q and j are alters, and $\sum_q p_{iq} \times m_{jq}$ measures the portion of i's relationship with j that is redundant to i's relationships with other primary contacts.

(5) $$\text{Maximum Degree} = L_{\max}$$

where $L_{\max}$ is the highest number of ties incident on a single node, not including the ego.

(6) $$\text{Mean Degree} = \frac{L_1 + L_2 + \cdots + L_N}{N}$$

where L is the number of ties from each node and N is the number of nodes, not including the ego.
