# Supplementary material for: Social networks and risk of delayed hospital arrival after acute stroke
Source: Nat Commun. 2019 Mar 14;10:1206. doi: 10.1038/s41467-019-09073-5 (PMC6418151; doi:10.1038/s41467-019-09073-5)
Supplement: Supplementary file 3 — Description of Additional Supplementary Files [file 41467_2019_9073_MOESM3_ESM.docx]

Description of Additional Supplementary Files

**Supplementary Movie 1:** A women experiences two similar stroke episodes in different social contexts that lead to diverging arrival times. In October 2016, a 70-year old woman had sudden onset of speech difficulty while riding in the car with a friend. On her friend’s insistence, she arrived at the hospital within 30 minutes. In December 2016, she again had sudden onset of speech difficulty while at her niece’s house. Her family asked if she was tired and advised her to go to sleep. She arrived at the hospital multiple days afterwards.
